# Effect of statin therapy on the progression of coronary atherosclerosis

**DOI:** 10.1186/1471-2261-12-70

**Published:** 2012-09-01

**Authors:** Jinwei Tian, Xia Gu, Yanli Sun, Xiang Ban, Yun Xiao, Sining Hu, Bo Yu

**Affiliations:** 1 Key Laboratories of Education Ministry for Myocardial Ischemia; Department of Cardiology, Second Affiliated Hospital of Harbin Medical University, Harbin, 150086, P.R. China; 2 Department of Pathology, Harbin Medical University, Harbin, China; 3 Department of Bioinformatics, Harbin Medical University, Harbin, China

**Keywords:** Atherosclerosis, Statin, Meta-analysis, Intravascular ultrasound

## Abstract

**Background:**

An increasing number of authors employing intravascular ultrasound (IVUS) and virtual histology (VH-IVUS) have investigated the effect of statin use on plaque volume (PV) and plaque composition. However, inconsistent results have been reported. Therefore, we conducted a meta-analysis to determine the appropriate regimen of statins to effectively stabilize vulnerable coronary plaques.

**Methods:**

Online electronic databases were carefully searched for all relevant studies. We compared mean values of PV and plaque composition between baseline and follow-up in patients receiving statin therapy. We pooled treatment effects and calculated mean differences (MD) with the 95% confidence interval (CI) using a random-effects model. By stratified analyses, we explored the influence of clinical presentation, dose and duration of statin treatment, and low-density lipoprotein-cholesterol (LDL-C) levels on the effects of statins.

**Results:**

Seventeen studies involving 2,171 patients were analyzed. Statin therapy significantly decreased PV (−5.3 mm^3^; 95% CI: –3.3 mm^3^ to −7.2 mm^3^; *P <* 0.001), without heterogeneity. When considering the dose and duration of statins used, only subgroups employing a high dose and long duration demonstrated a significant reduction in PV (*p <* 0.001). A significant decrease in PV was noted if achieved LDL-C levels were <100 mg/dL (*p <* 0.001). Statin treatment could induce a twofold decrease in PV in patients with acute coronary syndrome (ACS) compared with that observed in patients with stable angina pectoris (SAP). A regressive trend was seen for necrotic core volume (MD: –2.1 mm^3^; 95% CI: –4.7 mm^3^ to 0.5 mm^3^, *P =* 0.11). However, statin use did not induce a significant change for fibrotic, fibro-fatty, or dense calcium compositions.

**Conclusions:**

Our meta-analysis demonstrated that statin therapy (especially that involving a high dose and long duration and achieving <100 mg/dL LDL-C levels) can significantly decrease PV in patients with SAP or ACS. These data suggested that statins can be used to reduce the atheroma burden for secondary prevention by appropriately selecting the statin regimen. No significant change in plaque composition was seen after statin therapy.

## Background

Statin treatment is regarded as one of the most effective methods for the stabilization of vulnerable atherosclerotic plaques, and is associated with improvements in outcome in patients with coronary heart disease (CHD) [1,2]. Statins have a wide range of biologic effects, including a decrease in the level of low-density lipoprotein-cholesterol (LDL-C) and high-sensitivity C-reactive protein, as well as a modest increase in the level of high-density lipoprotein-cholesterol [3]. With respect to secondary prevention, statins are considered to be essential in subjects with CHD [4].

Outcomes have been shown to be improved after statin treatment, but the mechanism by which statins confer cardiovascular benefit is not precisely understood. Some angiographic studies have shown only minimal increases in lumen area in target lesions in patients administered statins [5]. Regression of coronary plaque volume (PV) and plaque composition as well as a reduction in plaque vulnerability are presumed to have important roles. One study employing a meta-analysis reported a significant reduction in plaque size after statin treatment [6], whereas no significant decrease in plaque size was shown by subgroup analyses according to follow-up time of statin use and LDL-C levels, suggesting that these results were not robust. An increasing number of intravascular ultrasound (IVUS) and virtual histology-intravascular ultrasound (VH-IVUS) studies have demonstrated that statin therapy can result in significant changes in PV and plaque composition [7,8]. However, not all studies have suggested that statin therapy can induce a significant reduction in the size and composition of plaques. These differences among studies may be due to different statin-administered strategies. Therefore, determining which regimen of statin administration is effective for stabilizing vulnerable coronary plaques is very important.

In the present study, we aimed to summarize the evidence on the effectiveness of statins on atherosclerosis development. We also sought to determine the extent to which such statin-induced changes in plaque composition was supported by evidence. We therefore undertook a meta-analysis to investigate if statin therapy can significantly change PV and plaque composition. By stratified analyses, we further explored the influence of clinical presentation, dose and duration of statin treatment, and LDL-C levels at follow-up upon the effects of statins.

## Methods

### Search strategies

The online electronic databases PubMed, EMBASE, and Cochrane Clinical Trials were searched carefully using the following terms: “Intravascular ultrasound” or “IVUS” and “HMG-CoA reductase inhibitor (s)”, or “statin(s)”, or “atorvastatin” or “pravastatin” or “simvastatin” or “cerivastatin” or “fluvastatin” or “lovastatin” or “mevastatin” or “pitavastatin” or “rosuvastatin”. The date range was 1990–2010 and there were no language restrictions. The literature search was accomplished independently by two well-trained reviewers. Discussions between the two reviewers were initiated if there were discrepancies in search results. Abstracts of conferences and recently published editorials were checked, as were the reference lists of identified articles and review articles.

### Inclusion criteria

The inclusion criteria for the meta-analysis were: (1) IVUS and/or VH-IVUS volume analyses at baseline and follow-up; (2) ≥1 statin therapy group.

### Exclusion criteria

Reviews, commentaries, editorials and case reports were not used. Studies analyzing other drugs, stents, graft disease, in-stent neointima and animal models were also excluded. We also excluded studies in which: there was no follow-up; not all patients were evaluated by IVUS or VH-IVUS; the volumetric parameters of plaques were not reported.

### Selection of studies

**Figure 1:**
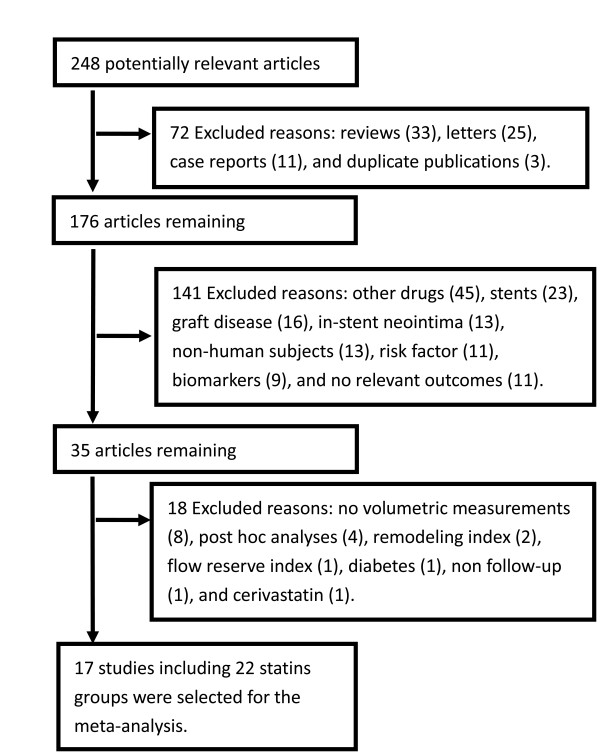
Study selection.

Two reviewers independently reviewed the articles. Study selection is summarized in Figure 1. Of 248 relevant studies, 213 were excluded (Figure 1). Of the remaining 35 potentially appropriate studies, 18 were excluded. Of these, 8 studies detected area measurements or did not provide absolute volume measurements; 4 carried out *post-hoc* analyses using experimental data from other studies which were included in our analyses; 2 measured the remodeling index ; 1 chose the coronary flow reserve index as the endpoint; 1 investigated patients with diabetes mellitus; 1 undertook IVUS at one time point; 1 considered cerivsatatin [9] (which was withdrawn from the market in 2001 due to its association with fatal rhabdomyolysis) only in the Discussion section. Consequently, 17 studies including 22 groups with 2,171 subjects were analyzed [10-26].

The main analysis of our study focuses on pre October 2010 papers but that since then 5 studies with 7 groups [27-31] were published whose principal findings were shown in the in Additional file 1: Table S1 and Additional file 2: Table S2 but were not included in the formal analysis.

### Data extraction

Two reviewers independently extracted the following variables: (i) first author's surname and year of publication of article; (ii) characteristics of the study population (sample size, age, sex, presentation); (iii) type and dose of statin; (iv) duration of follow-up; (v) LDL-C levels at baseline and follow-up; (vi) VH-IVUS volume data. Inconsistencies in the interpretation of data were discussed until a consensus was reached. If a study lacked complete data, the investigators of the primary study were contacted to provide information.

The methodological quality of the studies included in the meta-analysis was independently scored by two reviewers using a validated five-point scale created by Jadad et al. The scale consists of three items describing randomization (0–2 points), masking (0–2 points), and dropouts and withdrawals (0–1 points) in the report of a randomized controlled trial [20]. A score of 1 is given for each of the points described. An additional point is obtained if the method of randomization and/or blinding is given and is appropriate, whereas one point is deducted if it is inappropriate. Higher scores indicate better reporting.

### Endpoints

Before IVUS analyses, based on reproducible landmarks (e.g., a calcium deposit, stent edge or side branch), the same segment was identified in the IVUS run at baseline and at follow-up. IVUS analyses were undertaken once at baseline and at follow-up by the same independent experienced investigator who was blinded to the patient groups. Manual detection of the lumen contour and the media–adventitia interface was undertaken by an experienced analyst blinded to baseline clinical characteristics and baseline angiographic characteristics of the lesions. The external elastic membrane volume and lumen volume were calculated. The difference between these two values was defined as PV.

VH-IVUS uses IVUS radiofrequency data to classify an atherosclerotic plaque into four compositions: fibrous, fibro-fatty, dense calcium, and necrotic core. These compositions are assigned color codes of green, greenish yellow, white and red, respectively. Color-coded tissue maps are then constructed. Compositions within the plaque can be identified, as previously validated by preliminary *in-vitro* and *in-vivo* studies [32,33]. Fibrous tissue was marked in green, fibro-fatty in yellow, dense calcium in white and necrotic core in red colors on the VH-IVUS image. The absolute value of each plaque composition was also calculated automatically by the software.

We compared the mean values of PV and plaque composition between baseline and follow-up in patients receiving statin therapy. We further analyzed the effects of statin treatment on plaque size by clinical presentation, dose and duration of statin treatment, and LDL-C levels at follow up.

### Statistical analyses

We pooled treatment effects and calculated mean differences (MD) with a 95% confidence interval (CI) for all endpoints by using a random-effects model [34]. Heterogeneity was tested using the Cochran Q test. We considered the results for heterogeneity to be significant at *P <* 0.10 (two-sided). Inconsistency of treatment effects was calculated for assessing the percentage of total variance across studies that was due to heterogeneity rather than chance. Publication bias was estimated using funnel plots. A strongly asymmetric plot suggested the underlying presence of publication bias (i.e., whereby small studies reporting positive outcomes are more likely to be published than equally small studies reporting negative results). RevMan 5.0.23 was used for analyses of statistical data. *P <* 0.05 was considered significant. For assessing the potential impact of publication bias, we calculated the *fail-safe number* using the method of Rosenberg and Orwin's [35]. (The *fail-safe number* represents the number of studies needed to make *P >* 0.05.)

By stratified analyses, we explored the influence of clinical presentation, dose and duration of statin treatment, and LDL-C levels at follow-up on the effects of statins. Of the 22 groups, 5 groups investigated patients with acute coronary syndrome (ACS) and 13 groups investigated patients with stable angina pectoris (SAP). When considering the dose and duration of statin therapy, enrolled studies were divided into four subgroups: subgroup 1, ≤10 mg/day of atorvastatin or an equivalent dose of other statins and ≤6 months of follow-up; subgroup 2, ≤10 mg/day of atorvastatin or an equivalent dose of other statins and >6 months of follow-up; subgroup 3, >10 mg/day of atorvastatin or an equivalent dose of other statins and ≤6 months of follow-up; and subgroup 4, >10 mg/day of atorvastatin or an equivalent dose of other statins and >6 months of follow-up. When considering LDL-C levels at follow-up, all enrolled studies were divided into three subgroups: subgroup 1, ≤70 mg/dL of LDL-C; subgroup 2, 70–100 mg/dL of LDL-C; and subgroup 3, ≥100 mg/dL of LDL-C.

## Results

Of the 22 groups, atorvastatin was used in 8 groups, rosuvastatin in 3 groups, simvastatin in 3 groups, pravastatin in 3 groups, pitavastatin in 1 group, fluvastatin in 1 group, and different statins in 3 groups (Additional file 3: Table S3). Each group enrolled mostly men. Of the 22 groups, 5 groups investigated patients with ACS, and 13 groups investigated patients with SAP. The remaining 4 groups did not exclude patients with ACS. The sample size of the studies was 17–446 patients. The mean age of patients in the studies was 61 ± 3.5 years (range, 55–67 years). The mean LDL-C level at baseline was 128.6 ± 22.6 mg/dL (range, 71–158.3 mg/dL). The mean LDL-C level at follow-up ranged was 85.3 ± 13.8 mg/dL (range, 60.8–110.4 mg/dL). The duration of follow up was 1.5–24 months.

**Figure 2:**
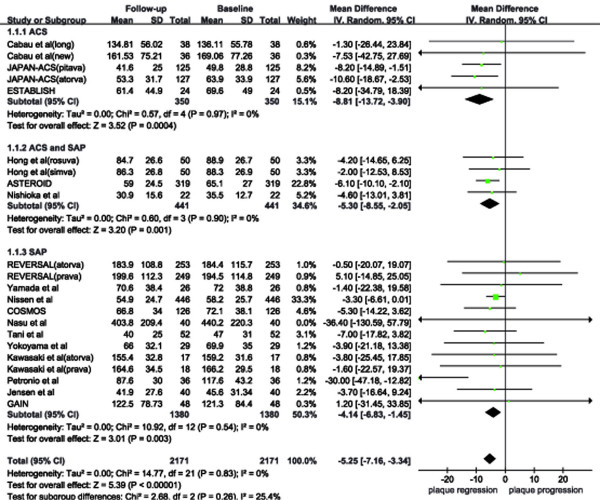
**Effect of statins on plaque volume. **Squares to the left of the vertical line indicate regression of coronary plaques, whereas squares to the right of the vertical line indicate progression of coronary plaques. The horizontal line through each square represents the 95% confidence interval (CI). The diamond represents the pooled effect (width of the diamond indicates the 95% CI). Abbreviations: ACS, acute coronary syndrome; SAP, stable angina pectoris.

**Figure 3:**
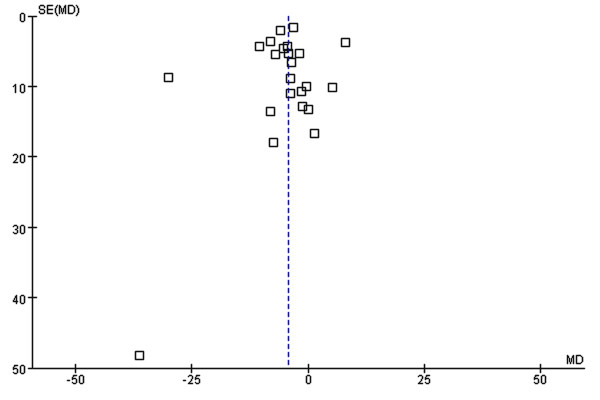
**Graphical representation of publication bias. **The dots, each representing one study, conform to a triangular shape: the publication bias is low.

The PV significantly decreased after statin therapy (MD: –5.3 mm^3^; 95% CI: –3.3 to −7.2 mm^3^; *P <* 0.001), without heterogeneity (*P =* 0.83 and *I*^*2*^*=* 0%; Figure 2). The funnel plot (Figure 3) did not show asymmetry consistent with publication bias. The *fail-safe N* was 142, suggesting that 142 additional ‘negative’ studies would be needed to negate our result. The decrease in PV in ACS patients was twofold than that observed in SAP patients (Table 1).

**Table 1:** Mean differences and 95% CIs in plaque volume achieved in patients taking one statin

**Subgroup**	**Number of studies**	**Number of patients**	***I***^***2 ***^**index**	**PV change (95% CI)**	***P***
All patients	19	2,171	0%	−5.3 mm^3^ (−7.2, –3.3)	<0.001
ACS patients	5	350	0%	−8.8 mm^3^ (−13.7, –3.9)	0.0004
SAP patients	13	1,380	0%	−4.1 mm^3^ (−6.8, –1.5)	0.003

Abbreviations: *ACS*, acute coronary syndrome; *PV*, plaque volume; *SAP*, stable angina pectoris; *I*^*2*^ index: degree of heterogeneity in the meta-analysis.

**Figure 4:**
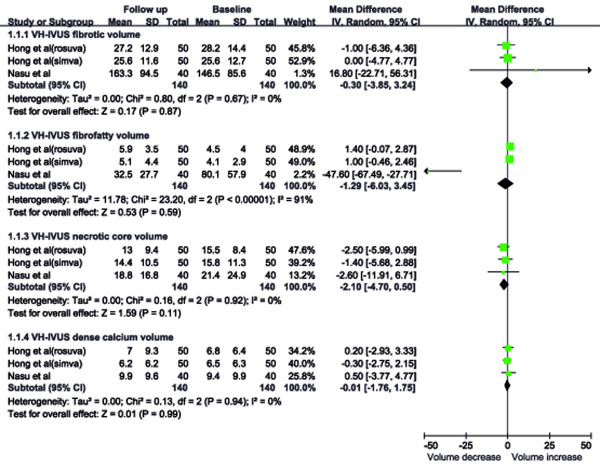
Effects of statins on plaque composition.

A regressive trend was seen for necrotic core volume (MD: –2.1 mm^3^; 95% CI: –4.7 mm^3^ to 0.5 mm^3^, Figure 4), but did not reach statistical significance (*P =* 0.11). No significant change was noted for the fibrotic, fibro-fatty, or dense calcium volume (*P =* 0.87, 0.59, and 0.99, respectively). Non-significant heterogeneity was detected for the fibrotic, necrotic core or dense calcium volume, but not for fibro-fatty volume (*I*^*2*^*=* 91%).

When considering the dose and duration of statin therapy, a significant decrease in PV was seen only in the studies involving administration of >10 mg/day of atorvastatin or an equivalent dose of other statins and >6 months of continuous therapy (MD, –5.2 mm^3^; 95% CI: –3.1 mm^3^ to −7.3 mm^3^; *P <* 0.001; *fail-safe N =* 45; 9 groups, 1,520 patients; Table 2), without heterogeneity.

**Table 2:** Stratified analyses by dose and duration of follow-up of statin therapy

**Subgroup**	**Number of studies**	**Number of patients**	***I***^***2 ***^**index**	**PV change (95% CI)**	***P***
1	3	99	0%	−5.4 mm^3^ (−13.8, 3.0)	0.21
2	5	415	59%	−7.8 mm^3^ (−20.2, 4.5)	0.21
3	2	41	0%	−5.6 mm^3^ (−22.3, 11.2)	0.52
4	9	1,520	0%	−5.2 mm^3^ (−7.3, –3.1)	<0.001

Abbreviations: *PV*, plaque volume. Subgroup 1: ≤10 mg/day of atorvastatin or an equivalent dose of other statins and ≤6 months of follow-up; subgroup 2: ≤10 mg/day of atorvastatin or an equivalent dose of other statins and >6 months of follow-up; subgroup 3: >10 mg/day of atorvastatin or an equivalent dose of other statins and ≤6 months of follow-up; subgroup 4: >10 mg/day of atorvastatin or an equivalent dose of other statins and >6 months of follow-up. *I*^*2*^ index: degree of heterogeneity in the meta-analysis.

When considering LDL-C levels at follow-up, significant decreases in PV were shown in studies with 70–100 mg/dL of achieved LDL-C levels (MD: –5.7 mm^3^; 95% CI: –2.7 mm^3^ to −8.6 mm^3^; *P <* 0.001; *fail-safe N =* 58; 13 groups, 1,372 patients; Table 3), and in the studies with <70 mg/dL of achieved LDL-C levels (MD: –5.9 mm^3^; 95% CI: –2.2 mm^3^ to −9.6 mm^3^; *P <* 0.001; *fail-safe N =* 5; 4 groups, 429 patients; Table 3). However, there was no a significant decrease in PV in studies with >100 mg/dL of achieved LDL-C levels (*P =* 0.15). There was no evidence of heterogeneity for the three subgroups.

**Table 3:** Stratified analyses by LDL-C levels at follow-up

**Subgroup**	**Number of studies**	**Number of patients**	**I**^**2 **^**index**	**PV change (95% CI)**	***P***
≤70 mg/dL of LDL-C	4	429	0%	−5.9 mm^3^ (−9.6, –2.3)	<0.001
70–100 mg/dL of LDL-C	13	1,372	9%	−5.7 mm^3^ (−8.6, –2.7)	<0.001
≥100 mg/dL of LDL-C	5	370	0%	−4.2 mm^3^ (−9.9, 1.5)	0.15

Abbreviations: *LDL-C*, low-density lipoprotein-cholesterol; *PV*, plaque volume. *I*^*2*^ index: degree of heterogeneity in the meta-analysis.

## Discussion

The present study demonstrated that statin therapy can significantly decrease the size of atherosclerotic plaques, whereas there were no significant changes in plaque composition. These findings are similar to the findings in some *in-vivo* human studies [36]. Our findings are supported by animal studies, which have demonstrated a reduction in plaque size and macrophage content as well as an increase in the amount of interstitial collagen in atherosclerotic lesions after lipid-lowering therapy (LLT) [37-39]. One study demonstrated that PV increased by 11.8% in the usual care group [25]. When considering the dose and duration of statin therapy, a significant decrease in PV was noted only in subgroups having a high dose and long duration of statin therapy. Based on achieved LDL-C levels, the present study demonstrated that a significant decrease in plaque size will occur only if LDL-C is <100 mg/dL. This finding supports the use of intensive LDC-L-lowering therapy in high-risk older subjects with established cardiovascular disease. These data indicate that coronary artery disease can be retarded (and even regress) if the favorable levels of LDL-C that were attained with statin therapy in the present study are achieved. Statin treatment induced a twofold decrease in PV in patients with ACS compared with that seen in patients with SAP, and this difference may originate from various plaque characteristics between the two cohorts [40]. Evidence suggests that patients with ACS have many greater-risk non-culprit plaques [41]. Multi-slice CT study has demonstrated that certain characteristics of vulnerable plaques (e.g., positive vascular remodeling and low plaque density) are more frequent in ACS lesions than in SAP lesions [42]. Some papers published since October 2010 further supported our conclusion that statin therapy can significantly retard or even decrease plaque progression in patients with coronary artery disease, especially when patients taking a high dose and long duration of statin regimen [27,29,31].

Plaque composition is now seen as being much more important than plaque size and the severity of stenoses. Plaques containing a soft lipid-rich core are particularly dangerous because such plaques are unstable and vulnerable to rupture, whereby highly thrombogenic plaques are exposed to blood flow [43,44]. Plaques that are prone to rupture have a large lipid core, occupy >40% of the total PV, and have a thin fibrous cap (diameter, <65 μm) [45]. Reduction in the size of the necrotic core might be one of several requirements for plaque stabilization. After statin therapy, the necrotic core tended to decrease in size, but this did not reach statistical significance. Some studies using optical coherence tomography (OCT) showed that the incidence of plaque rupture was significantly decreased and that the thickness of the fibrous cap tended to increase with statin therapy [46]. Based upon VH-IVUS findings, “fibro-fatty” is defined as fibrous tissue with significant lipid interspersed in collagen [32]. The interspersed lipids in fibro-fatty tissue might regress from the atherosclerotic intima with a reducing continued influx of lipid. Hence, an accumulation of interstitial collagen in fibrous tissue might be significantly increased by LLT. However, we failed to observe significant changes in fibro-fatty volume in the present study. In agreement with our conclusion, one recent study has demonstrated that statins does not lead to significant changes in plaque composition [28]. One explanation for those findings in the present study could be that the dose of statins selected in the three groups should be almost equivalent to 10–20 mg/day of atorvastatin, which is relatively low to be effective for stabilizing coronary plaques in CHD [13,14]. Further studies are needed to determine the effect of intensive statin therapy on plaque composition. Another reason for those findings in the present study could be low statistical power. For primary efficacy, i.e., change in necrotic core volume, a sample size of ≈238 patients was specified for 80% power and a two-sided α level of 0.025 to detect an expected change of −2 mm^3^ (assuming a standard deviation (SD) of 10 mm^3^). Indeed, a substantial residual risk of clinical events remains in most patients who have undergone secondary prevention despite the use of statins [47]. In the Scandinavian Simvastatin Survival Study (4S), therapy reduced the relative risk of coronary events by ≈30% over 5 years from an absolute risk of 28% to 19% [48,49]. Novel anti-atherosclerotic agents in addition to statins may be needed to more effectively stabilize plaque composition.

One study reported a non-significant decrease in PV in a subgroup with <100 mg/dL LDL-C levels at follow-up and in a subgroup with >6 months of statin therapy; these data were probably due to a relatively small sample size and non-consideration of the consequences of statin dose [6]. Based on the achieved LDL-C levels, our subgroup analyses demonstrated significant plaque regression at a follow-up LDL-C level of <100 mg/dL. Those results support the efficacy of Adult Treatment Panel III (ATP III) Cholesterol Guidelines and improve the practice of lipid lowering with statins [50]. Recently, one IVUS study have reported that a positive association was noted between the changes in LDL-C and plaque volume [30]. Some landmark studies have also shown that high dose and long-term lipid-lowering with statins may be better for reducing cardiovascular adverse events in patients with cardiovascular disease [51-53]. Previous analyses suggest that benefit can be achieved by treating older patients with CHD more aggressively to reduce LDL-C levels to <100 mg/dL [54]. Those benefits may be explained by the results in the present study which confirm that statin therapy at high dose over the long term are better for retarding and reversing atherosclerosis. The present study showed that, by appropriately selecting the regimen, statins can be used to reduce the atheroma burden for secondary prevention. The present study was based on large numbers of patients and had significant statistical power. No heterogeneity in treatment effects was observed for the main endpoints. Moreover, the funnel plot did not show asymmetry consistent with publication bias, and the *fail-safe N* for the main endpoints was relatively large. Additionally, our results were not influenced by including the results of the ENCORE II study using 0.2–0.8 mg/day of cerivastatin [9].

It has been suggested that statin administration can decrease plaque size, lipid content, and the thickness of the fibrous cap [55-57]. Nevertheless, the mechanisms by which statins contribute to plaque stabilization and atherosclerotic regression are incompletely understood. Recently, OCT, integrated backscatter intravascular ultrasound (IB-IVUS), VH-IVUS, and angioscopy have been shown to provide *in-vivo* real-time qualitative information of atheromas, such as lipid content, fibrous-cap thickness, calcification, macrophage infiltration, thrombosis, and rupture [58-60]. Therefore, investigators could design large-scale studies to elucidate the changes in the size and composition of plaques after LLT with statins by combining these catheter-based intravascular imaging tools.

Some researchers have paid close attention to the pleiotropic effects of statins that are independent of lipid lowering effects. These include immune modulation, inhibition of the proliferation and migration of smooth muscle cells, and inhibition of angiogenesis [61-65]. The primary action of statins is a cholesterol-dependent effect: inhibition of the synthesis of L-mevalonate (a precursor of cholesterol). The secondary actions are pleiotropic: inhibition of the synthesis of isoprenoids such as farnesyl pyrophosphate and geranylgeranyl pyrophosphate (which are essential for membrane translocation and the biological activity of members of the GTPase family) [66,67]. Williams *et al.* documented that pravastatin-treated monkeys had atherosclerotic lesions with less neovascularization and less infiltration of macrophages independent of plasma concentrations of lipoproteins [37]. Atorvastatin treatment has been shown to dose-dependently reduce the level of a serum marker of oxidative stress [68]. Furthermore, those pleiotropic effects of statins are often larger with high doses of statins, and there may be individual differences among different statins [69]. The present study has shown that regimens employing high doses of statin result in plaque regression in subjects with CHD. The dose-dependent pleiotropic effects of statins may be one of the explanations for our findings [70]. In the present study, greater benefit was noted among patients with ACS, which led to an improvement in outcome [71]. The pleiotropic effects of statins (particularly their anti-inflammatory and antithrombotic properties) have been invoked to explain the greater benefits seen in ACS [72,73]. However, it remains unclear which statin has the greatest and most rapid pleiotropic effects. Large, prospective, controlled studies are needed to determine the role of the pleiotropic effects of statins on atherosclerosis.

Our systematic review and meta-analysis had limitations. First, as is true with all systematic reviewed and meta-analyses, our study may have been affected by publication bias (though we did not find evidence of such bias). Second, the inclusion criteria of patients in primary studies (including those in the present study) were not standardized (e.g., LDL-C levels at baseline and clinical presentation). Third, although the studies included in this meta-analysis were homogeneous, variations in the dose, type, and duration of statin use among studies might have affected our results. Our random-effects model attempted to account for between-study variability, and the effects of this heterogeneity were examined.

## Conclusions

Statins are prescribed extensively for the treatment of hypercholesterolemia. Our meta-analysis demonstrated that statin therapy (especially at a high dose and of long duration) can induce a significant decrease in PV. Based on the achieved LDL-C levels, subgroup analyses demonstrated significant plaque regression at a follow-up LDL-C level of <100 mg/dL. We also showed that statin treatment can induce a twofold decrease in PV in patients with ACS compared with that seen in patients with SAP. However, no significant change in plaque composition was seen after statin therapy.

## Abbreviations

ACS: Acute coronary syndrome; CHD: Coronary heart disease; CI: Confidence interval; IVUS: Intravascular ultrasound; MD: Mean differences; LDL-C: Low-density lipoprotein-cholesterol; LLT: Lipid-lowering therapy; OCT: Optical coherence tomography; PV: Plaque volume; SAP: Stable angina pectoris; VH: Virtual histology.

## Competing interests

The authors declare that they have no competing interests.

## Authors’ contributions

BY conceived of the study idea, and BY, JT, XG, and YS contributed to the study design. XG conducted the literature review. JT, SH, and YX performed the data extraction. BY, YS, JT, and XB were involved in consensus agreements concerning data discrepancies. YX designed and YX and JT conducted the statistical analyses. XB, YX, and SH participated in interpretation of data. JT, BY, and XB drafted and revised the manuscript. All authors were involved in revising the article for important intellectual content, interpreting the data, and approved the final version to be published. All authors read and approved the final manuscript.

## Pre-publication history

The pre-publication history for this paper can be accessed here:

http://www.biomedcentral.com/1471-2261/12/70/prepub

## Supplementary Material

Additional file 1 **Table S1. **General characteristic of the 5 studies including 7 groups without formal analysis. Click here for file

Additional file 2 **Table S2. **(VH)-IVUS findings at baseline and follow-up in patients taking one statin from 5 studies without formal analysis. Click here for file

Additional file 3 **Table S3. **General characteristic of the included studies. Click here for file
