# Supplementary material for: Effect of statin therapy on the progression of coronary atherosclerosis
Source: BMC Cardiovasc Disord. 2012 Sep 1;12:70. doi: 10.1186/1471-2261-12-70 (PMC3468364; doi:10.1186/1471-2261-12-70)
Supplement: Additional file 1 — Table S1. General characteristic of the 5 studies including 7 groups without formal analysis. [file 1471-2261-12-70-S1.doc]

**Supplement Table 1 General characteristic of the 5 studies including 7 groups without formal analysis**

| Study | Clinical | Age (year) | Statins type | Dose (mg) | Duration (month) | LDL at Baswline (mg/dl) | LDL at Follow up (mg/dl) | Quality Score |
| --- | --- | --- | --- | --- | --- | --- | --- | --- |
| Kovarnik 2012 | CAD | 65.1 | Ato | 10 | 12 | 104.2±30.9 | 100.4±30.9 | 5 |
| Lee 2011 | CAD | 60.8 | Sim | 40 | 9 | 127.1±23.5 | NA | 5 |
| Hirayama 2011 | CAD | 59 | Ato | 10-20 | 20 | 146.2±28.8 | 87.9±15.8 | 3 |
| Hong 2011 | CAD | 59 | Ros | 20 | 11 | 122±37 | 62±20 | 5 |
| Hong 2011 | CAD | 58 | Ator | 40 | 11 | 124±117 | 70±24 | 5 |
| Nicholls 2011 | CAD | 57.9 | Ator | 80 | 26 | 119.9±28.9 | 70.2±1.0 | 5 |
| Nicholls 2011 | CAD | 57.4 | Ros | 40 | 26 | 120.0±27.3 | 62.6±1.0 | 5 |

Abbreviations: Ato, atorvastatin; CAD, coronary artery disease; LDL, low-density lipoprotein cholesterol; NA, data not available; Ros, rosuvastatin; Sim, simvastatin.
