# Supplementary material for: Effect of statin therapy on the progression of coronary atherosclerosis
Source: BMC Cardiovasc Disord. 2012 Sep 1;12:70. doi: 10.1186/1471-2261-12-70 (PMC3468364; doi:10.1186/1471-2261-12-70)
Supplement: Additional file 2 — Table S2. (VH)-IVUS findings at baseline and follow-up in patients taking one statin from 5 studies without formal analysis. [file 1471-2261-12-70-S2.doc]

**Supplement Table 2 (VH)-IVUS findings at baseline and follow-up in patients taking one statin from 5 studies without formal analysis.**

| **Group** | **Baseline (mm^3^)** | **Follow-up (mm^3^)** | **Percent change(%)** | ***P*** |
| --- | --- | --- | --- | --- |
| **Plaque Volume** | | | | |
| **Lee 2011 ( simvastatin)** | 170.6±63.6 | 175.9±46.9 | 5.3±42.9 | 0.36 |
| **Hirayama 2011 (atorvastatin)** | 158.0 ± 45.8 | 128.8 ± 31.5 | NA | <0.001 |
| **Hong 2011 (rosuvastatin)** | 166±93 | NA | -4.4±7.3 | >0.05 |
| **Hong 2011 (atorvastatin)** | 190±119 | NA | -3.6±6.8 | >0.05 |
| **Nicholls 2011(atorvastatin)** | 144.2±63.8 | 138.5±63.2 | NA | <0.001 |
| **Nicholls 2011 (rosuvastatin)** | 144.1±60.8 | 135.7±57.7 | NA | <0.001 |
| **Plaque Composition** | | | | |
| **Kovarnik 2012 (fibrotic volume)** | 132±78.6 | 125.6±76.0 | NA | >0.05 |
| **Kovarnik 2012 (fibrofatty volume)** | 50±35.1 | 42.4±36.2 | NA | >0.05 |
| **Kovarnik 2012 (necrotic volume)** | 28.1±27.7 | 36.6±32.4 | NA | >0.05 |
| **Kovarnik 2012 (calcium volume)** | 15.3±8.2 | 23.0±27.1 | NA | >0.05 |

Abbreviations: NA, data not available; SD, standard deviation.
