# Supplementary material for: Effect of statin therapy on the progression of coronary atherosclerosis
Source: BMC Cardiovasc Disord. 2012 Sep 1;12:70. doi: 10.1186/1471-2261-12-70 (PMC3468364; doi:10.1186/1471-2261-12-70)
Supplement: Additional file 3 — Table S3. General characteristic of the included studies. [file 1471-2261-12-70-S3.doc]

**Supplement Table 3 General characteristic of the included studies**

| Study | Clinical | Age (year) | Statins type | Dose (mg) | Duration (month) | LDL at Baseline (mg/dl) | LDL at Follow up (mg/dl) | Quality Score |
| --- | --- | --- | --- | --- | --- | --- | --- | --- |
| COSMOS 2009 | SAP | 62.6 | Ros | 16.9 | 19 | 140.2 ± 31.5 | 82.9 ± 18.7 | 3 |
| Cabau (long) 2009 | ACS | 60.0 | Statins | NA | 1.5 | 71.0 ± 23.0 | 77.0 ± 25.0 | 3 |
| Cabau (new) 2009 | ACS | 56.0 | Statins | NA | 1.5 | 100.0 ± 30.0 | 63.0 ± 17.0 | 3 |
| JAPAN-ACS, 2009 | ACS | 62.5 | Pit | 4 | 8-12 | 130.9 ± 33.3 | 81.1 ± 23.4 | 5 |
| JAPAN-ACS, 2009 | ACS | 62.4 | Ato | 20 | 8-12 | 133.8 ± 31.4 | 84.1 ± 27.4 | 5 |
| Nasu 2009 | SAP | 63.0 | Flu | 60 | 12 | 144.9 ± 31.5 | 98.1 ± 12.7 | 3 |
| Hong 2009 | # | 58.0 | Sim | 20 | 12 | 119.0 ± 30.0 | 78.0 ± 20.0 | 5 |
| Hong 2009 | # | 59.0 | Ros | 10 | 12 | 116.0 ± 28.0 | 64 .0± 21.0 | 5 |
| Nissen 2007 | SAP | 57.0 | Ato | 23 | 24 | 84.3 ± 18.9 | 87.2 ± 22.6 | 5 |
| Yamada 2007 | SAP | 66.7 | Ato | 11.5 | 12 | 123.0 ± 17.0 | 83.0 ± 22.0 | 3 |
| ASTEROID, 2006 | # | 58.5 | Ros | 40 | 24 | 130.4 ± 34.3 | 60.8 ± 20.0 | 3 |
| Tani et al. 2005 | SAP | 63.0 | Pra | 10-20 | 6 | 123.0 ± 28.0 | 104.0 ± 20.0 | 3 |
| Yokoyama 2005 | SAP | 62.1 | Ato | 10 | 6 | 133.0 ± 13.0 | 87.0 ± 29.0 | 3 |
| Kawasaki 2005 | SAP | 67.0 | Pra | 20 | 6 | 149.0 ± 19.0 | 102.0 ± 13.0 | 3 |
| Kawasaki 2005 | SAP | 66.0 | Ato | 20 | 6 | 155.0 ± 22.0 | 95.0 ± 15.0 | 3 |
| Petronio 2005 | SAP | 63.0 | Sim | 20 | 12 | 114.0 ± 21.0 | 94.0 ± 9.0 | 3 |
| Nishioka 2004 | # | 65.5 | Statins | NA | 6 | 132.0 ± 33.0 | 106.0 ± 20.0 | 2 |
| ESTABLISH, 2004 | ACS | 61.3 | Ato | 20 | 6 | 124.6 ± 34.5 | 70.0 ± 25.0 | 4 |
| Jensen 2004 | SAP | 57.7 | Sim | 40 | 12 | 158.3 ± 35.9 | 84.6 ± 21.9 | 3 |
| REVERSAL, 2004 | SAP | 56.6 | Pra | 40 | 18 | 150.2 ± 25.9 | 110.4 ± 25.8 | 5 |
| REVERSAL, 2004 | SAP | 55.8 | Ato | 80 | 18 | 150.2 ± 27.9 | 78.9 ± 30.2 | 5 |
| GAIN, 2001 | SAP | 60.7 | Ato | 32.5 | 12 | 155.0 ± 34.0 | 86.0 ± 30.0 | 4 |

Abbreviations: ACS, acute coronary syndrome; Ato, atorvastatin; Flu, fluvastatin; LDL, low-density lipoprotein cholesterol; NA, data not available; Pit, pitavastatin; Pra, Pravastatin; SAP, stable angina pectoris; Ros, rosuvastatin; Sim, simvastatin.

#indicates that this study includes patients with ACS or SAP.
